# Knowledge and adherence to isoniazid preventive therapy among people living with HIV in multilevel health facilities in South-East, Nigeria: baseline findings from a quasi-experimental study

**DOI:** 10.11604/pamj.2020.36.261.22496

**Published:** 2020-08-10

**Authors:** Ifeyinwa Chizoba Akamike, Ijeoma Nkem Okedo-Alex, Adaoha Pearl Agu, Chihurumnanya Alo, Lawrence Ulu Ogbonnaya

**Affiliations:** 1 Department of Community Medicine, Alex Ekwueme Federal University Teaching Hospital, Abakaliki, Ebonyi State, Nigeria,; 2 Department of Community Medicine, Ebonyi State University, Abakaliki, Ebonyi State, Nigeria

**Keywords:** Knowledge, adherence, isoniazid preventive therapy, people living with HIV

## Abstract

**Introduction:**

isoniazid preventive therapy is a crucial component of TB/HIV collaborative program and patient good knowledge and adherence to this preventive treatment are essential in improving implementation. The aim of this study was to determine the knowledge and adherence to isoniazid preventive therapy among patients receiving HIV care.

**Methods:**

this is a baseline result of a quasi-experimental study which was carried out among 200 patients receiving HIV care in six high patient load health facilities providing comprehensive HIV care in Ebonyi State. This included a tertiary health facility and five secondary level health facilities. We used structured interviewer-administered questionnaire to collect information from the participants. Adherence was assessed by self-reports. Descriptive, bivariate and multivariate logistic regression analyses were conducted using SPSS version 20 at 5% level of significance.

**Results:**

majority (65%) of the respondents were between 30 and 49 years and most (73.5%) were females. Majority (85%) had been on antiretroviral therapy (ART) for more than one year. More than half of the respondents had ever received and had been counselled on IPT (55%, 62% respectively) while only 17.5% were on IPT during the study. More than half (60.5%) of the respondents had low level of knowledge. Marital status was the only predictor of knowledge. Unmarried respondents were 2 times more likely to have knowledge of IPT compared with the married (AOR = 2.11, CI = 1.10-4.06). Among the 35 patients who were on IPT, 32 (91%) reported good adherence in the 30 days preceding the survey. **Conclusion:** there was poor knowledge of IPT among the respondents however self-reported adherence was high. We recommend intensification of general and personalized education of PLHIV on IPT by health workers.

## Introduction

An estimated 0.8% [0.6-0.9%] of adults aged 15-49 years worldwide are living with HIV [1]. The WHO African region bears the highest burden, with almost 1 in every 25 adults (4.1%) living with HIV and accounting for about two-thirds of the people living with HIV worldwide [1]. In 2017, 10 million people became ill with TB, and 1.6 million died from the disease (including 0.3 million among people with HIV) [2]. One of the major reasons for the increase in global burden of tuberculosis is the HIV pandemic [3-5]. Isoniazid preventive therapy (IPT) is recognised as an important component of collaborative TB and HIV activities to reduce the burden of TB in people living with HIV (PLHIV) [6]. The World Health Organization (WHO) recommends IPT as a part of the Three I´s for HIV/TB control which include: IPT, Intensified TB case finding, and Infection control for TB [7]. The current recommended dose of IPT for adults is 300 mg of isoniazid per day for six months [7]. Adults and adolescents living with HIV are to be screened with the symptom checklist for TB, so as to rule out active TB infection [7]. Those reporting one or more symptoms may have active TB and should be further evaluated and then either treated for TB or, once active TB is ruled out, started on IPT [8]. Those reporting none of the symptoms should be started on IPT [8]. Adherence is an active, deliberate and responsible process of care whereby the patient works to maintain health in collaboration with healthcare providers [9]. Patient non-adherence can take various forms; patient may misunderstand, perform wrongly, forget, or completely ignore the recommendation given to them by their healthcare professionals [4, 10]. Some studies have reported low level of knowledge of latent TB and the IPT guideline [5,11]. Studies have shown that adherence rates for Isoniazid Preventive Therapy vary significantly from 34%-98% [12-17]. Adherence can be improved if patients have good knowledge of the needs and benefits of IPT and this can be facilitated through counseling of these patients by healthcare workers [4, 18]. The study set out to determine the knowledge and adherence of patients to isoniazid preventive therapy.

## Methods

**Study area:** this study was carried out in HIV clinics of six health facilities in Ebonyi State. This included one tertiary and five secondary level health facilities. There are two tertiary health facilities in the state (Alex Ekwueme Federal University Teaching Hospital Abakaliki and National Obstetric Fistula Centre), owned by the Federal Government. Currently, there are 555 registered private and public health facilities in the state. These include 13 general hospitals, six mission hospitals, 417 primary health care centres and 119 private hospitals/clinics. However, in order to provide world-class health services, Ebonyi State Government is presently upgrading 171 PHCs i.e one PHC per ward and 13 General Hospitals i.e one General Hospital per LGA in the State [19]. Public, private and mission hospitals provide comprehensive HIV care in the state. Comprehensive HIV care includes: voluntary and confidential counseling and testing, prevention of HIV transmission, including mother to child transmission, prophylaxis against opportunistic infections, diagnosis and treatment of HIV related conditions including opportunistic infections, and antiretroviral treatment [20].

**Study population:** the study population comprised of PLHIV receiving care in the selected health facilities. Patients who completed IPT within the last two years prior to the onset of study were excluded.

**Study design:** this is a baseline survey of a quasi-experimental study that focused on improving the knowledge and adherence of health workers to the IPT guideline and the knowledge and adherence of patients to isoniazid preventive therapy guideline. This paper presents the baseline findings among the patients.

**Sampling technique:** simple random sampling was used to select high patient load (greater than or equal to 100 enrolled patients) health facilities from a list of health facilities that provide comprehensive HIV care in the State. Proportionate allocation of sample size was done to ascertain the sample size per health facility. Most of the health facilities run at least one clinic day in a week and patients are given at most two monthly appointments, therefore data was collected over a period of two months and all eligible patients were selected till the sample size was reached.

**Sample size determination:** the formula for comparing two proportions was used considering a significance level of 5%, desired power of 80%, P1 & P2 of 76.9% and 55.8% [21] respectively, with an attrition rate of 20% to arrive at 93. Data was collected from 100 respondents for each arm (intervention and control) of the quasi-experimental study. Data from the 200 respondents are presented in this paper.

**Data collection:** we collected data using structured interviewer administered questionnaire. The questionnaire was used to collect information on socio-demographic characteristics, knowledge about IPT, and adherence to IPT.

**Measurement of variables:** the independent variables include socio-demographic and clinical characteristics of patients such as age, gender, level of education, religion, employment status, duration on ART, duration of HIV diagnosis. The dependent variables include **patient knowledge of IPT:** five questions were used to assess knowledge of PLHIV about isoniazid preventive therapy. A correct answer for each was given a score of 2, and 0 was given for a wrong answer. The score varied from 0-10 points and was classified into two levels as follows: High level: 5- 10 scores; Low level: 0-4 scores. **Patient adherence to IPT:** this was measured by self-reported adherence to IPT in the 30 days preceding the survey. The following formula was used to calculate adherence rate: (Total number of doses of IPT taken)/(Total number of prescribed doses of IPT)*100. Adherence level of 80% and above was considered adherent and less than 80% as non-adherent.

**Statistical analysis:** data analysis was carried out using Statistical Package for Social Sciences (IBM-SPSS), Microsoft Window version 20 software. Descriptive statistics of the variables were presented using frequency tables, and relevant means, standard deviations, and proportions were calculated. The chi-square test was carried out to test for observed associations between variables. The level of significance was set at p < 0.05 and confidence interval at 95%. Patient knowledge of IPT was cross-tabulated with patient socio-demographic characteristics. Logistic regression was done to ascertain predictors of patient knowledge of IPT.

**Ethical consideration:** ethical approval was obtained from the research and ethics committee of Alex Ekwueme Federal University Teaching Hospital with REC approval number: 18/01/2017 - 09/03/2017.

**Confidentiality:** the data collection tool (questionnaire) did not include self-identifying characteristics such as participant´s name and contacts.

**Informed consent:** participants were informed of the purpose of the study and their roles and rights as participants. Their consent to participate was sought and documented using an informed consent form. A box was provided for participants´ to sign.

**Voluntary participation:** participation was voluntary and respondents were informed that they can withdraw from the study at any stage if they wish, without any adverse consequences to them.

**Risks:** participants were informed that there were no risks associated with participating in the study since there are no invasive procedures involved.

**Benefits:** participants were also informed that the findings of the study would help in the designing of programs and policies that would help improve patient care.

## Results

Table 1 shows that majority (> 65%) of the respondents were between 30 and 49 years and most (73.5%) were females. Slightly more than 5% of them were married and half had at least secondary education. Almost all the respondents (97.5%) were Christians, and majority (78%) were employed. Majority of the respondents had been diagnosed of HIV for one year and above while majority had been on ART for more than one year. Table 2 shows that greater proportions of the respondents were aware of IPT and had ever received counseling on IPT. Slightly more than half of the respondents had ever received IPT while only 17.5% were currently on IPT. Table 3 shows that less than half of the patients answered the knowledge questions correctly except for the question on the knowledge of isoniazid function which had 52.5% of the respondents answering correctly. Composite knowledge of the respondents is also shown on Table 3. More than half of the respondents had low level of knowledge. Marital status was found to be the only predictor of knowledge (Table 4). Unmarried respondents were 2 times more likely to have good knowledge of IPT when compared with the married (AOR = 2.11, CI = 1.10-4.06).

**Table 1:** socio-demographic and clinical characteristics of patients

Variable		
	N=200	
**Age Group(years)**	n(%)	n(%)
<30	33	16.5
30-39	70	35.0
40-49	60	30.0
≥50	37	18.5
**Mean age (mean &plusmn;SD)**	39.45&plusmn;10.28	
Gender		
Male	53	26.5
Female	147	73.5
**Marital status**		
Married	124	62.0
Others	76	38.0
**Level of education**		
Primary education and less	100	50.0
Secondary education and more	100	50.0
**Religion**		
Christianity	195	97.5
Others@	5	2.5
**Employment Status**		
Employed	156	78.0
Unemployed	44	22.0
**Duration of diagnosis**		
< 1 year	24	11.0
≥ 1 year	176	89.0
**Duration on ART**		
≤1 year	30	15.0
>1 year	170	85.0

**Table 2:** awareness, counseling and use of IPT among patients

Variable	Frequency	Percent
	n=200	(%)
**Awareness of IPT**		
Yes	132	66.0
No	68	34.0
**Ever Counselled on IPT**		
Yes	124	62.0
No	76	38.0
**Ever Been on IPT**		
Yes	110	55.0
No	90	45.0
**Currently on IPT**		
Yes	35	17.5
No	165	82.5
**IPT Adherence**	n=35	
Yes	32	91.4
No	3	8.6

**Table 3:** knowledge of isoniazid preventive therapy among patients

	N=200	
Proportion of respondents with correct answers to knowledge questions		
	Correct	(%)
Knowledge of isoniazid function	105	52.5
Knowledge of IPT service availability	97	48.5
Knowledge of drug name for IPT	22	11.0
Knowledge of duration of treatment	84	42.0
Knowledge of who should receive IPT	50	25.0
Composite Knowledge of patients		
Knowledge level	N=200	(%)
Low level	121	60.5
High level	79	39.5

**Table 4:** predictors of knowledge of IPT

Independent variable	Adjusted odds ratio	95% CI for AOR		Pvalue
		**Lower**	**Upper**	
**Age**				
>35	1.19	0.64	2.22	0.57
≤35	1			
**Duration on ART**				
>1 year	0.54	0.23	1.28	0.16
≤1 year	1			
**Marital status**				
Others	2.11	1.10	4.06	0.03*
Married	1			
**Gender**				
Female	0.79	0.39	1.60	0.51
Male	1			
**Educational level**				
Secondary or more	1.21	0.67	2.17	0.53
Primary or less	1			
**Employment status**				
Unemployed	0.98	0.47	2.04	0.93
Employed	1			
*Statistical significance				

## Discussion

This study assessed the knowledge and adherence to isoniazid preventive therapy among PLHIV receiving care in HIV clinics in Ebonyi State. More than 50% of respondents had ever been counselled on IPT and were aware of IPT. This finding differs from what was reported in another study in Ethiopia where majority (70.2%) were not aware of IPT [5]. This finding of high awareness and counseling may have effect on patient knowledge since counselling serves as a way of providing information to the receiver. Majority of the respondents had been on ART for more than a year and so would have attended the clinics many times which gives the respondent the opportunity of being counselled. In health facilities, patient counseling is a routine activity carried out especially in the clinics and may be the reason for the high proportion of those that had ever been counselled and also the high level of awareness.

The proportion of patients who were on isoniazid preventive therapy was low (17.5%). The low uptake of IPT corroborates what was reported in a tertiary health facility in south eastern Nigeria which found that 30% of patients were started on IPT [22]. This finding is also similar to another study in Ethiopia which reported that 32% of patients were provided with IPT [5]. The finding also disagrees with a study carried out in Thailand where uptake of IPT was found to be 88.5% [23]. The explanation for the high level of uptake in the Thailand study may be the preparation and training of the health workers that was done prior to the survey.

Knowledge of isoniazid preventive therapy was low among the patients as about 60% of them had low level of knowledge. This finding agrees with the study in Ethiopia which found that only 29.8% of respondents had some information on IPT [5]. Patient adherence rate to IPT in this study was optimal with almost 100%adherence rates. This high level of adherence corroborates the finding of another study that was carried out in a tertiary health facility in Ebonyi state which revealed that adherence to IPT was 95.7% [24]. This similarity is not surprising since both studies were carried out in the same setting. Several other previous studies have also reported high levels of adherence to IPT [12, 17, 18]. The good adherence to therapy observed in this study may be because these patients were already on antiretroviral therapy and quality adherence counseling for the antiretroviral drugs is part of the routine activities for HIV care and therefore could influence adherence to any other treatment given a longside the ARDs. Marital status was the only predictor of IPT knowledge among the patients in this study. People who were not married were more likely to have high level of knowledge than those who were married. This finding may be explained by the fact that unmarried people are likely younger than the married ones and younger people have the capacity to retain knowledge more than those that are older. One of the strengths of this study is the fact that it was conducted in more than one health facility and also involved both the tertiary and secondary level facilities. One drawback however is that self-reported adherence has limitations in that it is not verifiable and may introduce recall bias.

## Conclusion

This study revealed a low level of knowledge of isoniazid preventive therapy among patients, despite high levels of awareness and counseling. It also showed that there is low uptake of IPT among the patients. However, among the patients that were on IPT, majority had good adherence which is commendable. We therefore recommend that proper education of the patients by health workers on the benefits of IPT should be emphasized. Additionally, further studies should be carried out to identify cost-effective interventions that will help improve knowledge.

### What is known about this topic

- Adherence level is high among PLHIV;
- High awareness of isoniazid preventive therapy among PLHIV.

### What this study adds

- Low level of in-depth knowledge about isoniazid preventive therapy and its benefits;
- Uptake of isoniazid preventive therapy among PLHIV is low.
